# Molecular characterization of proliferative lupus nephritis

**DOI:** 10.1186/ar4648

**Published:** 2014-09-18

**Authors:** Samir V Parikh, Huijuan Song, Jay Vance, Jianying Zing, Lianbo Yu, Ana Malvar, Brad H Rovin

**Affiliations:** 1 Ohio State University Medical Center, Columbus, OH, USA; 2 Hospital Fernandez, Buenos Aires, Argentina

## Background

The molecular events occurring within the kidney at diagnosis of lupus nephritis (LN) are incompletely understood. Despite use of intense immunosuppression, response rates are disappointingly low. We postulate that characterizing these events will lead to biomarker discovery and guide therapeutics. As proof of concept we evaluated the molecular pathology of LN kidney biopsies in a Latino cohort.

## Methods

We studied 19 pairs of proliferative LN biopsies and four normal control biopsies using Nanostring RNA technology. All patients had at least two biopsies with the first at LN flare and the second after a median follow-up of 8 months. Based on clinical parameters, five patients achieved complete remission (CR), 10 partial remission (PR) and four had no response (NR) by the time of repeat biopsy. A panel of 511 immune-response genes was analyzed for each biopsy. Data were log_2 _transformed and quartile normalization was used to normalize data across samples. A linear model was used to compare the four groups (normal/CR/PR/NR) at baseline and a paired *t *test was used to compare baseline expression data at baseline and follow-up for each patient. Transcripts were considered to be differentially expressed if they differed by at least twofold and if *P *< 0.01.

## Results

At flare, three transcripts were uniquely expressed in CR, seven transcripts in PR, and 20 transcripts in NR when compared with normal. When compared with NR flares, CR flares showed increased MME, FADD, and CD274 and decreased ITGB2 and C1S expression. Change in transcript expression from initial to repeat biopsy was compared within each group and between groups. From biopsy 1 to biopsy 2, the CR group showed increased NCAM1 and decreased ILRL1 and FKBP5 expression. The NR group showed increased IL1RAP and CD5 and decreased C7, IL28B, and IL12RB1 expression. When compared with the CR group, the NR group showed increased IL17a, NOS2, FCAR and IL1RAP and decreased NCAM1, C7, and IL28B expression. NR flares had significant activation of T-cell and B-cell receptor signaling, IL-10, STAT3, and TNF-R pathways when compared with CR flares. Compared with NR flares, CR flares showed significant activation of T-cell regulation, antigen presentation, and apoptotic pathways.

## Conclusion

The results of this study suggest that the molecular characteristics of responders are different than nonresponders. These data imply that molecular analysis of LN biopsies may provide prognostic information and guide choice of therapy.

